# SWE and SMI ultrasound techniques for monitoring needling treatment of ankylosing spondylitis: study protocol for a single-blinded randomized controlled trial

**DOI:** 10.1186/s13063-021-05344-z

**Published:** 2021-06-07

**Authors:** Mengyu Wang, Wen Fu, Lingcui Meng, Jia Liu, Lihua Wu, Yingjun Peng, Ziping Li

**Affiliations:** 1 The Second Clinical College of Guangzhou University of Chinese Medicine, 111 Dade Rd, Guangzhou, 510120 Guangdong Province China; 2 The First Affiliated Hospital of Henan University of Chinese Medicine, 19 Renmin Rd, Zhengzhou, 450004 Henan Province China; 3 The Second Affiliated Hospital of Guangzhou University of Chinese Medicine, 111 Dade Rd, Guangzhou, 510120 Guangdong Province China; 4 Shenzhen Bao’an Traditional Chinese Medicine Hospital, Guangzhou University of Chinese Medicine, 25 Yu’an 2nd Rd, Shenzhen, 518000 Guangdong Province China

**Keywords:** Acupuncture, Ankylosing spondylitis, Musculoskeletal ultrasound, Physical dysfunction, Randomized controlled trial

## Abstract

**Background:**

Ankylosing spondylitis (AS) is a high-incidence disease in young men that interferes with patients’ physical and mental wellbeing and overall quality of life (QoL). It is often accompanied by arthralgia, stiffness, and limited lumbar flexibility. Acupuncture is safe and effective for reducing the symptoms of AS, but the underlying mechanisms by which it does so are not fully understood. Therefore, to objectively assess acupuncture efficacy, which is critical for patients making informed decisions about appropriate treatments, we will use shear-wave elastography (SWE) and superb microvascular imaging (SMI) ultrasound techniques to evaluate elasticity of lumbar paraspinal muscles and blood flow to the sacroiliac joint (SIJ) in AS.

**Methods:**

We will recruit a total of 60 participants diagnosed with AS and 30 healthy subjects. Participants will be randomly allocated 1:1 to either an acupuncture group or a sham control acupuncture group. Primary-outcome measures will be musculoskeletal ultrasound, Ankylosing Spondylitis Quality of Life Scale (ASQoL), Bath Ankylosing Spondylitis Metrology Index (BASMI), and the Visual Analogue Scale (VAS) for pain. Secondary outcome measures will be the Bath Ankylosing Spondylitis Disease Activity Index (BASDAI), Bath Ankylosing Spondylitis Function Index (BASFI), and Fatigue Scale-14 (FS-14). We will monitor the effect of acupuncture or sham acupuncture on blood flow and SIJ inflammation using SMI, lumbar-muscle stiffness using SWE and the lumbar paraspinal-muscle cross-sectional area (CSA) using a two-dimensional (2D) grayscale imaging. QoL, physical function, and fatigue will be assessed using an evaluation scale or questionnaire developed for this study, with outcomes measured by the ASQoL, BASMI, BASDAI, BASFI, and FS-14. Healthy subjects will not receive acupuncture but undergo only musculoskeletal ultrasound at baseline. Acupuncture and sham control acupuncture interventions will be conducted for 30 min, 2–3 times/week for 12 weeks. Musculoskeletal ultrasound will be conducted at baseline and post-intervention, while other outcomes will be measured at baseline, 6 weeks, and post-intervention. The statistician, outcome assessor, and participants will be blinded to treatment allocation.

**Discussion:**

The results of this single-blinded, randomized trial with sham controls could help demonstrate the efficacy of acupuncture and clarify whether musculoskeletal ultrasound could be used to evaluate AS.

**Trial registration:**

ClinicalTrials.gov ChiCTR2000031476. Registered 3 April 2020.

## Background

Ankylosing spondylitis (AS) is a systemic disease characterized by chronic inflammation of the axial joint, involving the sacroiliac joint (SIJ), with primary clinical symptoms of arthralgia, stiffness, and limited flexibility [1]. Over the past 15years, the total prevalence of AS in the mainland China has been 0.29%, ranging from 0.42% in males to 0.15% in females [2]. The primary pathology includes inflammation of the bony attachments of tendons, ligaments, or joint capsules [3]. Physical dysfunction in AS can potentiate serious health conditions and lead to fatigue, depression, anxiety, and decreased quality of life (QoL) [4, 5]. The disease imposes substantial physical and social burdens on patients and can interfere with work and schooling [6, 7]. The goals of treatment are to alleviate symptoms, improve functioning, decrease disease complications, and forestall skeletal damage as much as possible [8]. The use of non-steroidal anti-inflammatory drugs (NSAIDs) and very expensive biologics is recommended for treatment [9]. However, due to the significant side effects [10], development of therapeutic resistance to drug treatment [11] and high drug costs, such use places a huge burden on patients. Thus, nonpharmacological intervention has become one of the most important options for AS [12]. Alternative therapies play an important role in treating this disease; for example, a combination of the Traditional Chinese Medicine (TCM) with, especially, acupuncture can achieve better effect.

Acupuncture is a safe alternative therapy with minimal side effects that has shown benefits in alleviating symptoms, reducing complications, and accelerating recovery in AS [13], and it has been shown to improve joint functional flexibility when used in the management of pain and rheumatic diseases [14–17]. Previous systematic studies support that acupuncture could be an option for treating AS [18, 19].

The 2019 guidelines of the American College of Rheumatology (ACR) [8] state that magnetic resonance imaging (MRI) is not suitable for detecting subclinical inflammation in patients with stable disease and recommend against patients obtaining spine radiographs at scheduled intervals to monitor progression, as the practice entails radiation exposure and will not lead to alteration of treatment in most cases. Therefore, it is particularly important to find a method that is safe and can be used at short intervals to detect subclinical inflammation in a timely manner. Ultrasound has become part of the fundamental Outcome Measures in Rheumatology (OMERACT) methodology validation by repeatedly exercises across various domains, including inflammatory burden and structural damage [20, 21]. Musculoskeletal ultrasound is a reliable evaluation method that directly visualizes characteristics of rheumatic conditions such as synovitis, tenosynovitis, bursitis, enthesitis, crystal depositions, bone erosions, or osteophytes/enthesophytes. It is more sensitive than X-ray or MRI in physical tendons, ligaments, or joint capsules [22, 23]. Early detection of subclinical lesions can help decision makers improve the quality of their daily clinical practice in rheumatic diseases.

Although musculoskeletal ultrasound is less sensitive than MRI and X-ray in early detection of synovitis and tenosynovitis [24], it is suitable for short-term evaluation of stable rheumatic diseases, offering highly accessible, low-cost, real-time imaging without radiation [25, 26]. The OMERACT Ultrasound Working Group has engaged in the validation of ultrasound as an outcome measurement instrument (OMI) by defining ultrasound manifestations of spondyloarthritis (SpA) [27].

This high-quality, randomized controlled trial (RCT) was designed via a pragmatic trial approach to objectively assess the efficacy of acupuncture using musculoskeletal ultrasound and a sham acupuncture group.

### Objectives

Our research hypotheses are as follows: (1) musculoskeletal ultrasound will be used as a short-term effective-examination method and objective auxiliary-examination method for AS. (2) Acupuncture will reduce subjective pain rating, improve joint functional flexibility, and the effect of pain on ASQoL.

## Methods/design

### Study setting

**Fig. 1:**
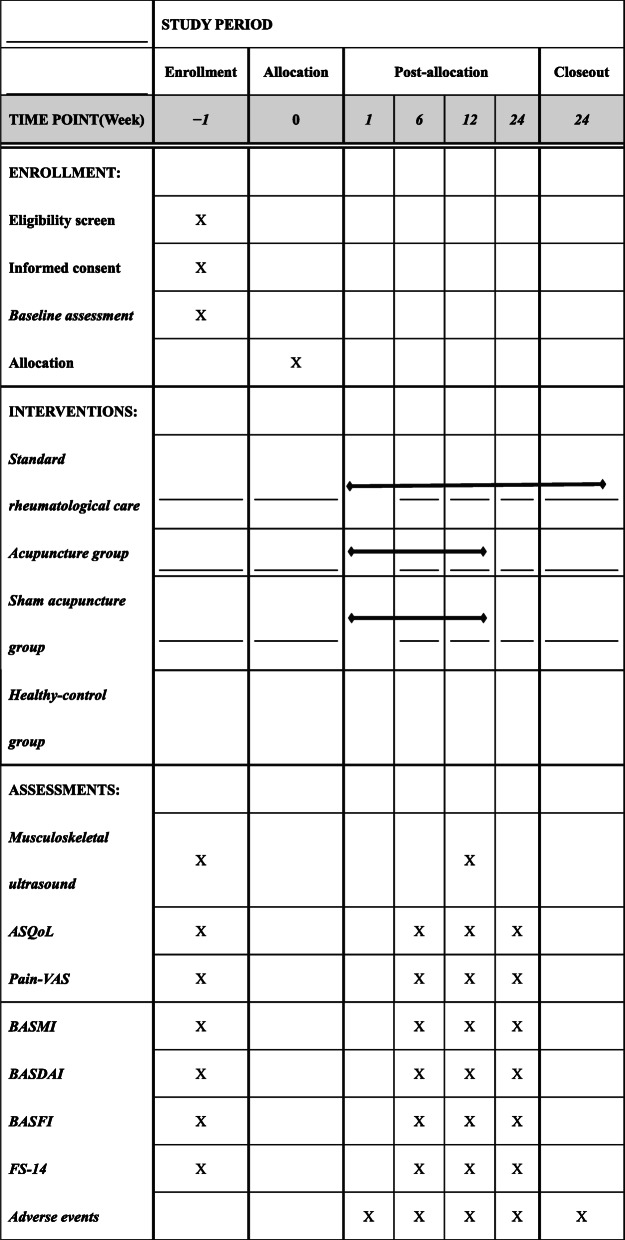
SPIRIT figure for schedule of enrollment, interventions and assessments. ASQoL, Ankylosing Spondylitis Quality of Life; VAS, visual analog scale; BASMI, Bath Ankylosing Spondylitis Metrology Index; BASDAI, Bath Ankylosing Spondylitis Disease Activity Index; BASFI, Bath Ankylosing Spondylitis Functional Index; FS-14, Fatigue Scale-14.

The study will follow the principles of the Consolidated Standards of Reporting Trials (CONSORT) for a two-arm randomized, superiority, parallel control clinical trial, as well as the Standards for Reporting Interventions in Clinical Trials of Acupuncture (STRICTA) statement for acupuncture [28, 29]. This will be a single-blinded RCT with a sample size based on published evidence in comparative studies. Sixty AS patients will be randomly established at a 1:1 ratio, including the acupuncture group and sham control acupuncture group. At the same time, thirty healthy volunteers will be recruited as blank control group. The sham control acupuncture group is set for placebo control to evaluate the clinical efficacy of acupuncture. AS patients blinded to study conditions. The study will be conducted at the Guangdong Provincial Hospital of Chinese Medicine (GPHCM), Department of Acupuncture, Guangdong, China, following the Standard Protocol Items: Recommendations for Interventional Trials (SPIRIT). For the participant timeline, see Fig. 1.

### Recruitment

A total of 60 AS patients age 18–60 years and 30 healthy volunteers will be recruited for this study. AS participants will be recruited from both the outpatient and inpatient departments of the GPHCM, Department of Rheumatology. Study flyers, bulletin boards at the hospital, and online advertisements will also be used for patient recruitment. Healthy volunteers will be recruited from among students of the Guangzhou University of Chinese Medicine. Treatment and measurements will be performed at the GPHCM. Participants will contact the recruitment staff by telephone or WeChat. Recruitment staff will be responsible for the enrollment of participants. If they meet the study criteria, they will be invited to the Department of Acupuncture and Moxibustion, Guangdong Provincial Hospital of Chinese Medicine, to undergo the study. Eligible candidates will be asked to sign an informed consent form before intervention begins.

### Inclusion criteria

Recruitment conditions are as follows: (1) a definite diagnosis of axial AS during a stable disease period; (2) 18–60 years of age, onset age <40 years; (3) currently being treated at a stable dose of medication for ≥4 weeks prior to randomization and have received no biologic therapy within the past 3 months; (4) course of disease ≤10 years; and (5) willingness to sign the informed-consent form. Potential participants who satisfy the inclusion criteria will be sent a more detailed information leaflet for informed consent. They will be contacted few days later to determine whether they are interested in participating, and, if so, an appointment will be made for them to visit the Guangdong Provincial Hospital of Chinese Medicine (GPHCM).

### Exclusion criteria

Potential participants will be excluded for the following reasons: (1) clinically important fracture of the spine; (2) spinal deformity or disability; (3) blood coagulation disorder; (4) presence of viral hepatitis, human immunodeficiency virus (HIV), or other blood infection; (5) pregnancy or lactation; (6) previous history of stroke or transient ischemic attacks; (7) pacemaker or other electrical device implanted; or (8) lack of consent, active pursuit of compensation or with pending litigation.

### Dropout criteria

1. Patients who have severe adverse reactions after acupuncture and cannot successfully complete the course of treatment;
2. The participant was unable to follow the protocol treatment for personal reasons during the course or use other traditional Chinese medicine therapies.

### Randomization

A computer algorithm [30] generated a permuted block randomization sequence that will allocate participants to either the acupuncture or sham control acupuncture group to ensure balanced group sizes and allocation concealment. We will use opaque, sealed envelopes in sequential order to contain allocation information.

### Blinding

As this is a single-blinded trial, patients will not know which treatment approach they will undergo. During the data collection and analysis stages, the clinical researcher, assessor, and statistician will not share study information with each other. Blinding will be assessed after the last treatment using a questionnaire that asks participants if they were in the real treatment group or the sham treatment group. The possible responses are “real treatment group,” “sham group,” or “do not know.”

### Intervention

All practitioners in this trial are licensed TCM acupuncture therapists with at least 5 years’ clinical experience, and they will be trained to master the study protocol. The acupuncturist will be asked to administer the sham intervention as they would administer standard manipulation, with the same enthusiasm. All participants will continue to receive standard rheumatological care.

### Acupuncture group

The acupuncture intervention program was designed by a senior acupuncturist. The acupoints will be *Shenshu* (BL23), *Ganshu* (BL18), *Yanglingquan* (GB34), *Jizhong* (DU6), *Jinsuo* (DU8), *Mingmen* (DU4), and *Yaoyangguan* (DU3). We will use sterile, disposable stainless-steel needles 0.3 mm in diameter, and 25 or 40 mm in length, depending on the acupoints. After eliciting the Deqi response, the researcher will apply electro-acupuncture by connecting an acupoint nerve stimulator (HANS-200A) to *Shenshu* (BL23) and *Ganshu* (BL18) at a frequency of 2 Hz for 30 min. Electro-acupuncture intensity will be set according to the maximum intensity tolerated by each subject (0.9–3.0 mA). Other needles will be stimulated manually every 10 min. All needles will be left in place for 30 min.

### Sham control acupuncture group

The acupuncture points will be lateral to those in the *verum* acupuncture group. However, instead of using a 0.25-mm diameter, 25-mm long, sterile disposable stainless-steel needles inserted into acupoints 2–3-mm deep without manipulation and into non-acupoints, we will include NP1 (1 cm outward horizontally of *Shenshu* [BL23]), NP2 (*Ganshu* [BL18] level outward by 1 cm), NP3 (1 cm behind *Yanglingquan* [GB34]), NP4 (1 cm to the right horizontally of *Jizhong* [DU6]), NP5 (1 cm to the right horizontally of *Jinsuo* [DU8]), NP6 (1 cm outward horizontally of *Mingmen* [DU4]), and NP7 (1 cm outward horizontally of *Yaoyangguan* [DU3]). Next, electro-acupuncture will be applied to *Shenshu* (BL23) and *Ganshu* (BL18; 0.1–0.3 mA). All needles will be left in place for 30 min. The inclusion of a placebo group will allow for comparison of active acupuncture care effects with manifestations of either sham treatment or psychic solace.

### Health control group

No acupuncture intervention will be conducted in healthy controls.

### Outcomes

#### Primary outcomes

Primary outcomes will be musculoskeletal-ultrasound, Ankylosing Spondylitis Quality of Life Scale (ASQoL) results, pain severity, and Bath Ankylosing Spondylitis Metrology Index (BASMI) results. Musculoskeletal ultrasound will use two-dimensional (2D) grayscale, shear-wave elastography (SWE) and superb microvascular imaging (SMI) techniques. The 2D grayscale technique will capture muscle thickness changes in the paraspinal and multifidus muscles. Changes in lumbar-muscle thickness can reflect muscle morphological change due to acupuncture treatment for somatic disorders. The lumbar paraspinal muscles play important roles in movement and control of the spine; studies have shown an inverse relationship between lumbar paraspinal-muscle CSA and lower-back disability but not between lumbar paraspinal-muscle CSA and pain intensity, suggesting that treatment strategies directed at increasing paraspinal-muscle size might be effective in reducing lower-back disability [31]. Later, a blinded tester will measure thickness at the levels of the L4–5 zygapophyseal joints using onscreen calipers [32]. SWE is an ultrasound technique that characterizes tissue mechanical properties based on the propagation of remotely induced shear waves [33]. It provides semiquantitative (color map) and quantitative (absolute SWE value) imaging biomarkers that are useful in assessing the elasticity of tendon and muscle composition and stiffness [34, 35] and in helping to distinguish between asymptomatic and symptomatic [36], with diseased tendons being significantly softer than healthy ones [37]. SMI is an ultrasound technique for vascular and microvascular examination. It can diagnose diseases associated with angiogenesis in their early phases and has value in grading disease activities and monitoring therapeutic responses [38]. SMI uses an intelligent algorithm that efficiently separates low-speed flow signals from motion artifacts and successfully extracts clinically relevant information [39]. We will test AS participants using SMI, record resistance index (RI), peak systolic velocity (PSV), and end diastolic velocity (EDV) to reflect SIJ inflammation in this trial. Ultrasound tests will be performed at baseline and week 12.

The ASQoL, Pain-Visual Analogue Scale for pain (VAS), and BASMI scales have anchors of 0 (none) to 10 (severe). Participants will evaluate their conditions over the preceding week. ASQoL records the impact of AS on health-related QoL from the patient’s perspective in terms of sleep, mood, motivation, coping, activities of daily living, independence, relationships, and social life [40]. It is a fixed-response questionnaire that asks endorsement (yes/no) of 18 items, and the total score is the sum of each score. Higher scores reflect low QoL. Regarding reliability, Cronbach’s α is reported between 0.89 and 0.92 in the different study groups. Regarding validity, ASQOL correlates moderately well with other AS-specific health outcome measures.

The Pain-VAS is selected to quantitatively evaluate the overall pain level of the body. BASMI [41] is associated with QoL, physical function, and psychological status and reflect the change of lumbar side flexion sensitive and reproducible. It is a composite score based on 5 direct measurements of spinal mobility [42]: lateral lumbar flexion, tragus-to-wall distance, lumbar flexion, intermalleolar distance, and cervical rotation angle, take the mean of the left and right measurements. Add together the scores for each item. Divide this by 5 to give the final BASMI score. The higher the BASMI score, the more severe the patient’s limitation of movement due to their AS. BASMI reliability has been shown to be good. Regarding construct validity, the BASMI has been shown to discriminate between patients with and without radiographic change due to AS.

#### Secondary outcomes

Secondary outcomes will be Bath Ankylosing Spondylitis Disease Activity Index (BASDAI), Bath Ankylosing Spondylitis Function Index (BASFI), Fatigue Scale-14 (FS-14) results. The BASDAI and BASFI were developed in 1994 using the Visual Analog Scale (VAS) [43, 44]. The BASDAI is a patient-generated index measuring disease activity in patients with AS. The index includes 6 items reflect levels of back pain, fatigue, peripheral joint pain and swelling, localized tenderness, and the duration and severity of morning stiffness. Visual analog scale (VAS, 0–10 cm) anchored by adjectival descriptors “none” and “very severe,” stiffness is anchored by a time scale (0–2 or more hours). The scores for questions 5 and 6 (severity and duration of morning stiffness) are averaged, and the result is then averaged with the remaining 4 question scores to give a final score out of 10. The total scores range from 0 (no disease activity) to 10 (maximal disease activity), and a cut off of 4 is used to define active disease. Internal consistency is good with a Cronbach’s α of 0.84–0.87 [45]. For validity, the BASDAI correlated well with the earlier Bath Disease Activity Index with no significant differences in score distribution, reproducibility, or sensitivity. The BASFI is a patient self-report questionnaire, includes 10 items to measures patients’ functional ability in terms of bending, reaching, changing position, standing, turning, and climbing steps. The overall index score is the sum of the individual scores, with higher scores reflecting greater functional impairment. Internal consistency is excellent, with Cronbach’s α reported at 0.936 [46]. And have good evidence of validity through comparison with instruments that measure similar or related constructs, and with measures of mobility. The FS-14 measures severity of physical and mental fatigue during their usual daily activities over the past week, which correlates positively with fatigue severity in AS. The FS-14 is a short questionnaire that asks endorsement (yes/no) of 14-item, easy to administer tool, and with good reliability and validity.

#### Other outcomes

Participant characteristics of age, weight, body mass index, current medical issues, current medications, and back pain history will be collected using electronic case report forms. Participants will be asked for number and types of medications taken, medication scheduling, and the dose used to self-manage AS symptoms at baseline and at weeks 6 and 12.

### Adverse events

Common treatment-related adverse events include dizziness, local subcutaneous hematoma, continuous post-needling pain, localized infections, and palpitations. Adverse events data will document the occurrence, duration, and severity of adverse reactions (symptoms and signs), and how the event was resolved (or not) during the treatment. Adverse events will be categorized by acupuncturists and related specialists as treatment-related or not within 24h after occurrence. If a participant reports a severe adverse event, it should be reported to principal investigators and the Institutional Ethics Committee (IEC) of Guangdong Provincial Hospital of Chinese Medicine, and they will decide to unblind and withdraw from the study. According to the situation, they will be referred to the emergency department or receive appropriate treatment. We will collect, assess, and report any spontaneously described adverse events from participants.

### Follow-up

Follow-up will occur 12 weeks after completion of the treatment program. This time point was selected to assess sustained long-term effectiveness of the intervention.

**Fig. 2:**
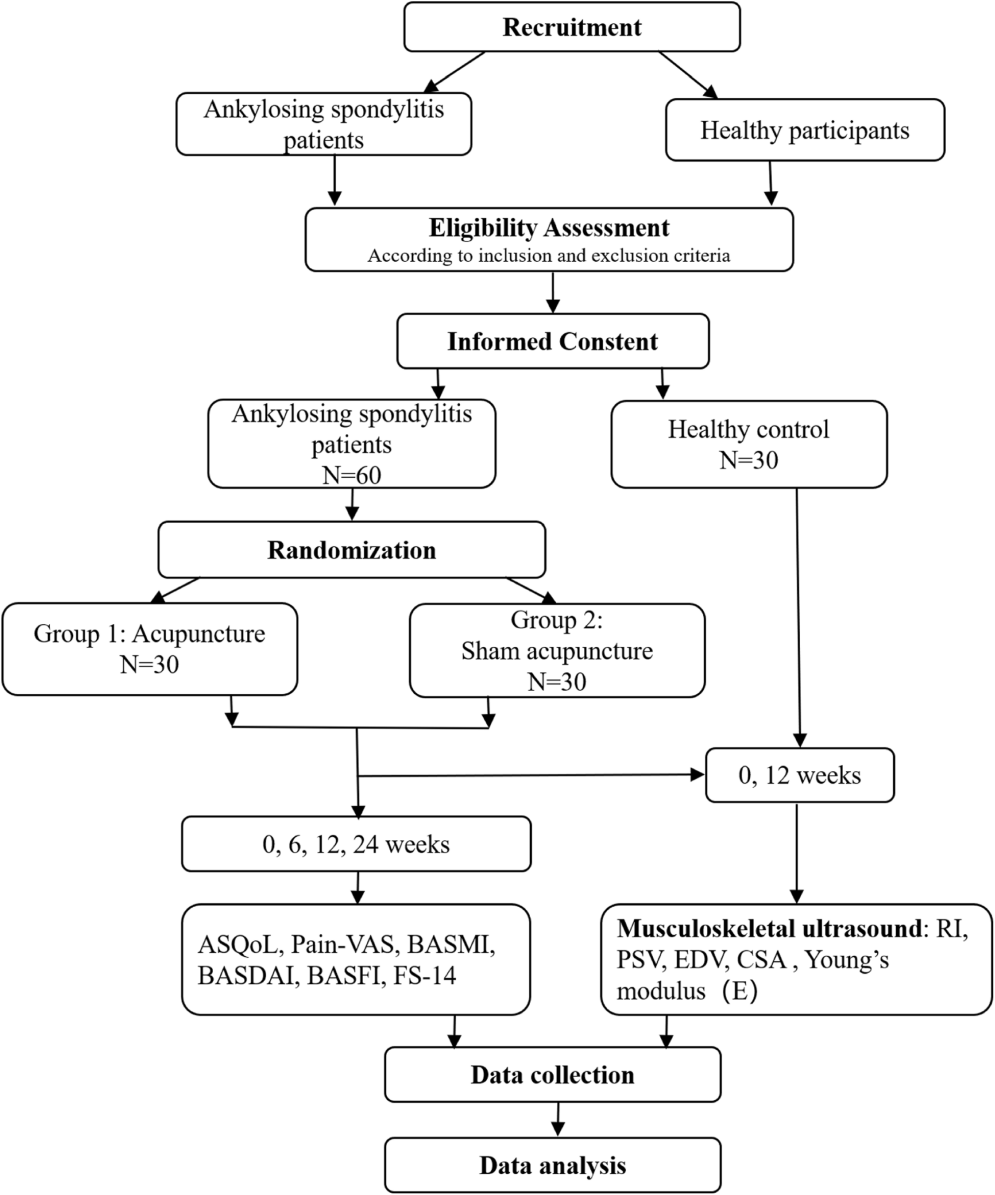
Trial work plan. Primary-outcomes measures will be musculoskeletal-ultrasound, ASQoL, Pian-VAS, and BASMI results. The remaining outcome measures will be for secondary outcomes. Follow-up will be performed at 24 weeks

The trial work plan is summarized in Fig. 2.

### Sample size

The required sample size for this study was estimated based on a study by You et al. [47]: at the week 24, pain VAS score was 3.87±1.54 (mean ± standard deviation [SD]) in needle-knife group and 4.59±1.57 in the control group. With a conservative estimate of 0.36point difference on a 10-point VAS score between the two arms, and assuming a standard deviation of 0.72 for both arms, based upon a two sample t tests assuming equal variance analysis conducted by an independent statistician via PASS 15.0, a sample size of 23 per group will yield a power of 90% at an alpha (α) value of 0.05. To account for an anticipated dropout rate of 20%, finally, the sample size was inflated to 60 with 30 patients per arm will be sufficient to detect a clinically important difference in this trial.

### Data management and monitoring

The case report form (CRF) includes observation time points, outcome measures, adverse events, and safety evaluations. The outcome assessors will be required to follow the requirements of the CRF and fill in the relevant information in a timely and accurate manner. The data collection and entry will be performed independently by two of the staff members and finalized by a third staff member. The main investigators, acupuncturist, sonographers, and laboratory staff will not be involved in data collection. Data will be obtained from the case record form (CRF), and only members of the trial team have access to the CRFs and perform double-data entry. The paper data will be saved with identification codes in seal. The electronic data will be stored in the ResMan Research Manager. Anonymized trial data will be shared with other researchers at least 3 years after the article is published. If reviewers or readers have any questions regarding our published data, they can contact the corresponding author for access to original data or visit ResMan (http://www.medresman.org/uc/project/projectadd.aspx). We will be monitored by the IEC of Guangdong Provincial Hospital of Chinese Medicine, and it is independent from the investigators and sponsor which will audit trial conduct every 12 months. And any modifications and corrections to operation procedures will be fully documented using a breach report form, monitored, and submitted to the directors of the ethics committee and China Clinical Trial Registration.

### Data analysis

We will use SPSS software version 18.0 (IBM Corp, Armonk, New York, US) to perform data analysis. Demographic and baseline data will be analyzed with standard descriptive statistics. Data will be presented as the mean ± standard deviation (SD). Between-group differences will be tested using repeated-measure analyses of variance (ANOVAs). The entire data analysis process will be performed by statisticians who are independent from the research team and blinded to the group settings. The accepted level of significance for all analyses will be *P* < 0.05.

### Ethics and dissemination

All candidates who agree to participate and who meet all of the inclusion criteria and none of the exclusion criteria will be provided informed consent to obtain full understanding of what study participation will entail and the potential risks. Participants have the right to discontinue participation at any time. Data will be used in the aggregate only, and no identifying characteristics of individuals will be published or presented. On the consent form, participants will be asked if they agree to use of their data should they choose to withdraw from the trial. Participants will also be asked for permission for the research team to share relevant data with people from the regulatory authorities, where relevant. This trial does not involve collecting biological specimens for storage.

The study conforms to the principles of the Declaration of Helsinki. Ethical approval has been obtained from the IEC of the Guangdong Provincial Hospital of Chinese Medicine (YF2019-232-01). The trial protocol has been registered at the Chinese Clinical Trial Registry (ChiCTR2000031476). Results will be disseminated through journal articles, a master’s thesis, or conference presentations. Data will be used in the aggregate only, and no identifying characteristics of individuals will be published or presented.

## Discussion

The purpose of this trial is to assess the impact of acupuncture in the management of AS. It is clear that increased muscle stiffness is associated with poor range of motion [48], and a chronic inflammatory condition has significant impact on QoL. Patients report that back pain, arthralgia, and stiffness influence their daily work productivity and nocturnal sleep quality [49]. Acupuncture is used to reduce muscle stiffness and increase somatic activity in AS patients, and it is monitored by musculoskeletal-ultrasound techniques that, if less operator dependent, could obtain more-stable and reliable results [50]. The design of an appropriate control group for a clinical trial is critical. We will set a healthy-control group for baseline comparison, as there is currently no standardized for musculoskeletal-ultrasound measurement of AS. However, it is difficult to use placebo needles for controls. Therefore, we will use non-acupoints and superficial puncturing, which are considered ineffective, in the sham acupuncture group.

NSAIDs are often first-line treatment for AS, and biologics are the next step up. However, patients who have inadequate response to NSAIDs but cannot afford biologics often experience significant pain and impairment of their QoL [51]. This trial would provide evidence for acupuncture care for this group of patients.

Despite the measures taken to ensure a well-controlled trial, there are several methodological limitations to this study. The treatment intervention duration of 12 weeks might make it difficult to ensure patient compliance. Other aspects such as the trial results are meant to describe an approach to treatment for the typical patient and cannot anticipate all possibilities; clinicians must make careful clinical assessments based on sound clinical judgment of each patient’s circumstances and consideration of the patient’s preferences. In spite of its limitations, we hope that the trial will help provide new insights into the value of acupuncture and the evidence of musculoskeletal-ultrasound measures in AS.

### Trial status

This trial is currently recruiting participants. Participants will be recruited for this study started in June 2020 and expected finish in December 2021. The protocol version number is 2019103103, date January 14, 2020.

## Data Availability

Not applicable.

## Abbreviations

AS: Ankylosing spondylitis
QoL: Quality of life
SWE: Wave elastography
SMI: Superb microvascular imaging
SIJ: Sacroiliac joint
BASMI: Bath Ankylosing Spondylitis Metrology Index
ASQoL: Ankylosing Spondylitis Quality of Life Scale
BASDAI: Bath Ankylosing Spondylitis Disease Activity Index
BASFI: Bath Ankylosing Spondylitis Function Index
FS-14: Fatigue scale-14
SAS: Self-Rating Anxiety Scale
SDS: Self-rating depression scale
CSA: Cross-sectional area
NSAIDs: Non-steroidal antiinflammatory drugs
TCM: Traditional Chinese Medicine
ACR: American College of Rheumatology
MRI: Magnetic resonance imaging
OMERACT: Outcome Measures in Rheumatology
OMI: Outcome measurement instrument
SpA: Spondyloarthritis
RCT: Randomized controlled trial
CONSORT: Consolidated Standards of Reporting Trials
STRICTA: Standards for Reporting Interventions in Clinical Trials of Acupuncture
GPHCM: Guangdong Provincial Hospital of Chinese Medicine
SPIRIT: Recommendations for Interventional Trials
HIV: Human immunodeficiency virus
2D: Two-dimensional
RI: Resistance index
PSV: Peak systolic velocity
EDV: End diastolic velocity
VAS: Visual analog scale
SD: Standard deviation
ANOVAs: Analyses of variance
CRF: Case report form
IEC: Institutional Ethics Committee
